# Supplementary material for: Tunneling time probed by quantum shot noise
Source: Nat Commun. 2018 Nov 22;9:4940. doi: 10.1038/s41467-018-07369-6 (PMC6250673; doi:10.1038/s41467-018-07369-6)
Supplement: Supplementary file 1 — Supplementary Information [file 41467_2018_7369_MOESM1_ESM.pdf]

Supplementary Information to: Tunneling time probed by quantum shot noise

P. Février[1] and J. Gabelli[1,2]

[1] *Laboratoire de Physique des Solides, CNRS, Université Paris-Sud, Université Paris-Saclay, Orsay, France*
[2] *Corresponding Author:* `julien.gabelli@u-psud.fr`

SUPPLEMENTARY NOTE 1
SAMPLE FABRICATION AND EXPERIMENTAL SETUP

The sample is a planar aluminum tunnel junction $SiO^{(20)}/Al^{(5)}/Al_20_3^{(2)}/Al^{(5)}/SiO^{(10)}$ deposited on a sapphire substrate. Numbers stand for the thickness in nm. The $100 \times 100\,\mu\mathrm{m}^2$ junction is fabricated by thin film deposition through shadow masks in a typical base pressure of 10^{-9} mbar with an oxidation of the first Al electrode in an oxygen glow discharge. The Kretschmann configuration is realized by using a $BK7$ glass prism [1]. The $I(V)$ characteristics is measured using a standard four points technique with a dc voltmeter whereas the bias-dependence of the tunneling conductance is measured using a standard lock-in technique. The current noise in the zero frequency limit is measured using a cross-correlation technique and a real time FFT-based spectral measurement performed with a digitizer in the frequency range $20 - 100\,\mathrm{kHz}$. Radiated power at wavelength λ is measured with filtered Si (at $\lambda = 0.9\,\mu\mathrm{m}$) and InGaAs (at $\lambda = 1.3\,\mu\mathrm{m}$) amplified detectors and a lock-in technique by modulating the voltage bias at 7 Hz. Their noise equivalent power are $\sim 1.2 \times 10^{-14}\,\mathrm{W}/\sqrt{\mathrm{Hz}}$ and $\sim 6.0 \times 10^{-14}\,\mathrm{W}/\sqrt{\mathrm{Hz}}$ respectively. To collect as much light as possible, the emitted light is refracted on a conical prism then collimated on the photon detector by using an aspherical lens (focal length $f = 8\,\mathrm{mm}$, numerical aperture $\mathrm{NA} = 0.5$). The detection efficiency is estimated at 80%.

SUPPLEMENTARY NOTE 2
CALIBRATION OF THE SHOT NOISE MEASUREMENT SETUP

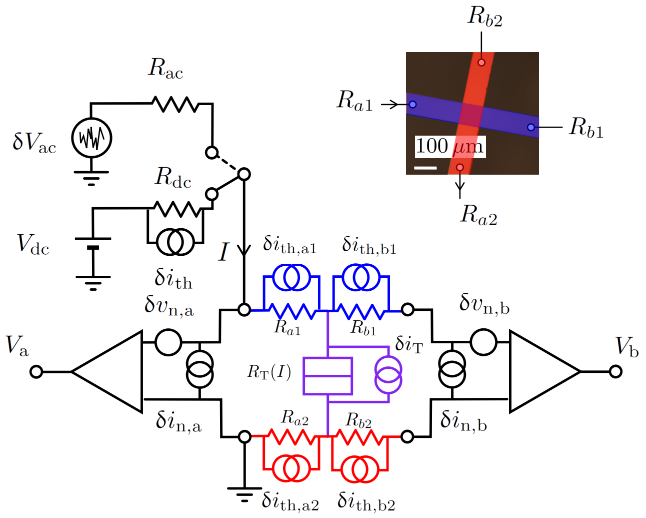

Supplementary Figure 1. Detailed view of the experimental setup used for the current noise measurement in the zero frequency limit. *Inset*: optical micrograph of the metallic cross-junction including the resistive electrodes R_{a1},R_{a2},R_{b1} and R_{b2} .

The current noise in the zero frequency limit is measured using a cross-correlation technique to remove the voltage noise of the amplifiers ($\delta v_n \simeq 2\,\mathrm{nV}/\sqrt{\mathrm{Hz}}$). If the current noise ($\delta i_n \simeq 15\,\mathrm{fA}/\sqrt{\mathrm{Hz}}$) can be neglected, the thermal noise of the contact resistances in series with the tunnel junction has to be subtracted. The resistance of the thin electrodes (5 nm) are indeed of the same order of magnitude than the differential tunnel resistance $R_{\mathrm{T}} \simeq 150\,\Omega$ at high voltage bias: $R_{\mathrm{a1}} = 221\,\Omega$, $R_{\mathrm{a2}} = 280\,\Omega$, $R_{\mathrm{b1}} = 172\,\Omega$, $R_{\mathrm{b2}} = 314\,\Omega$ (inset of Supplementary Figure 1). The voltage noise $S_{\mathrm{vv}}(V) \equiv \langle V_{\mathrm{a}} V_{\mathrm{b}} \rangle$ measured by the experimental set up depicted in Supplementary Figure 1 is:

$$S_{\mathrm{vv}}(V) = g\left[|Z_{\mathrm{setup}}(V)|^2 S_{\mathrm{ii}}(V) + S_{\mathrm{vv,setup}}(V)\right], \quad (1)$$

where g is the global gain of the amplifier chain, Z_{setup} is the transimpedance and $S_{\mathrm{vv,setup}}$ the excess noise related to the measurement setup. Supplementary Figure 2 shows the voltage noise spectral density S_{vv} measured in the frequency range $[20\,\mathrm{kHz}, 100\,\mathrm{kHz}]$. It cannot be directly compared to the current noise spectral density S_{ii} of the tunneling current because of the voltage dependence of $R_{\mathrm{T}}(V)$. A white voltage noise source δV_{ac} is then used to calibrate the detection setup. If δV_{ac} is high enough to neglect the intrinsic noise of the junction, the measured voltage

noise $S_{\rm vv,cal}$ enables to determine $Z_{\rm setup}$ and $S_{\rm vv,setup}$

$$|Z_{\rm setup}(V)|^2 = \frac{1+R_{\rm a}/R_{\rm dc}}{1+R_{\rm a}/R_T(V)} \frac{S_{\rm vv,cal}/g}{(\delta V_{\rm ac}/R_{\rm ac})^2}, \tag{2a}$$

$$S_{\rm vv,setup}(V) = \frac{2k_{\rm B}T_{300\rm K}}{R_{\rm dc}} \left(1 + \frac{T/T_{300\rm K}}{1+R_{\rm T}(V)/R_{\rm a}}\right) \frac{S_{\rm vv,cal}/g}{(\delta V_{\rm ac}/R_{\rm ac})^2}, \tag{2b}$$

where $R_{\rm dc} = 10\,{\rm k}\Omega$, $R_{\rm ac} = 10\,{\rm M}\Omega$, $R_{\rm a} = R_{\rm a1} + R_{\rm a2} \simeq 500\,\Omega$, $T_{300\rm K} = 300\,{\rm K}$ and T is the temperature of electrons. Supplementary Figure 3 shows the current shot noise and the theoretical expectation given by the FDR (section IV). The thermal noise measured at zero voltage bias for different temperatures is shown on the inset of Supplementary Figure 3 and is in good agreement with the fluctuation-dissipation theorem $S_{\rm ii}(eV=0) = 2k_{\rm B}T/R_{\rm T}$. Note the typical temperature dependence of the tunneling junction resistance which increases when the temperature decrease [2]. As mentioned in the article, the Joule heating ($P_{\rm J} \sim 5\,{\rm mW}$) cannot explain the discrepancy between the data and the theory. The electron-phonon coupling for $T \geq 100\,{\rm K}$ gives a thermal conductance $G_{\rm e-ph} \geq 10^{17}{\rm W.m^{-3}.K^{-1}}$ which leads to an electronic temperature equals to the temperature T_0 of the lattice such as: $T - T_0 < 0.5\,{\rm mK}$ [3]. One indeed deduces that the temperature of electrons is homogeneous over the whole sample.

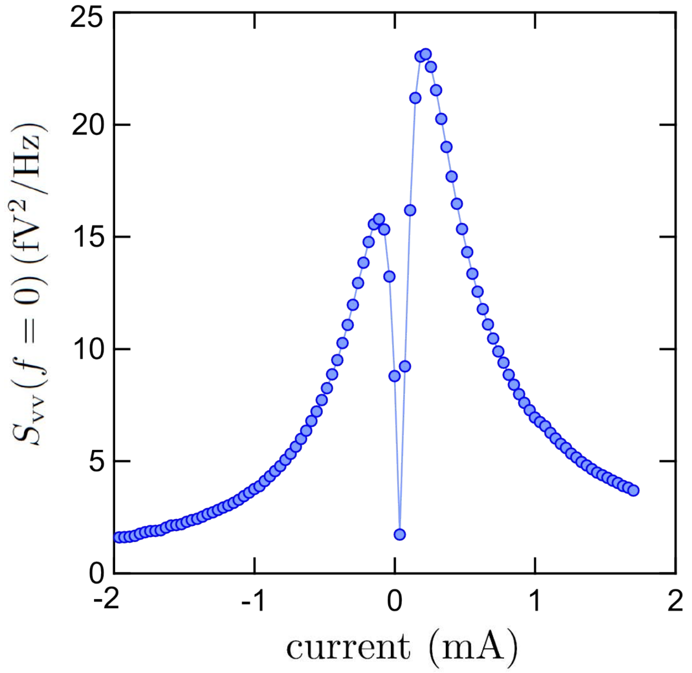

Supplementary Figure 2. Voltage noise spectral density measured in the bandwidth [10 kHz, 100 kHz].

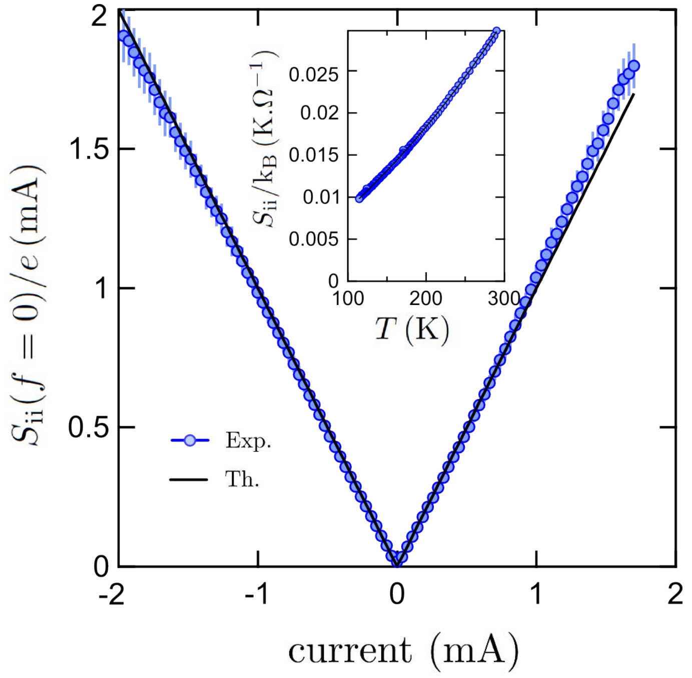

Supplementary Figure 3. Current noise spectral density measured in the bandwidth [10 kHz, 100 kHz]. Solid line corresponds to the fluctuation-dissipation theorem expectation. *Inset*: thermal noise measured at zero voltage bias for different temperatures.

SUPPLEMENTARY NOTE 3
ELECTRONIC TRANSPORT IN A TUNNEL JUNCTION IN THE FAR FROM EQUILIBRIUM REGIME

Energy and voltage dependence of the transmission - $I(V)$ characteristics for a 3D tunnel junction

We consider a tunnel junction with the surface area S and a large number of transverse channels labeled by the wave vector $\mathbf{k}_\perp = (k_\mathrm{x}, k_\mathrm{y})$. We assume that electrons are scattered elastically on the tunneling barrier without any inelastic energy loss inside the barrier. The tunnel current I is then given by the Landauer-Büttiker formula:

$$I(V) = \frac{2e}{h}\int_0^{+\infty} d\epsilon \sum_{k_\mathrm{x},k_\mathrm{y}} \mathcal{T}_{3\mathrm{D}}(\epsilon, k_\mathrm{x}, k_\mathrm{y})\left[f(\epsilon) - f(\epsilon + eV)\right], \tag{3}$$

where $f(\epsilon) = [1 + \exp((\epsilon - \epsilon_\mathrm{F})/k_\mathrm{B}T)]^{-1}$ is the Fermi-Dirac distribution, ϵ_F the Fermi energy and $\mathcal{T}_{3\mathrm{D}}(\epsilon, k_\mathrm{x}, k_\mathrm{y})$ the transmission probability for an incoming electron with a transverse wave vector $\mathbf{k}_\perp$ and a total energy $\epsilon = \frac{\hbar^2 k_\parallel^2}{2m} + \epsilon_\parallel$. The factor 2 accounts for the spin degeneracy.

We follow the Simmons' model ([4, 5] of the supplementary) where electrons are assumed to be free particles which have no interaction with each other and with the lattice. It allows us to describe electrons by one-electron wave function with a wave vector $\mathbf{k} = \left(k_\mathrm{x}, k_\mathrm{y}, k_\parallel\right)$ and an energy ϵ:

$$\epsilon = \frac{\hbar^2}{2m}|\mathbf{k}|^2 = \frac{\hbar^2}{2m}\left(k_\mathrm{x}^2 + k_\mathrm{y}^2 + k_\parallel^2\right) \tag{4}$$

A free electron moves in an energy potential which only depends on longitudinal direction $\parallel$. The Schrödinger equation can be splitted into three independent 1D problems: one for each dimension. The total wave function is then the product of one-dimensional solutions. The solutions for transversal directions (x and y direction) have the form $\psi_\perp^\pm = \exp(\pm i\ \mathbf{k}_\perp \cdot \mathbf{r}_\perp)$, which are independent of z. This is consistent with the conservation of the component of the vector parallel to the barrier. The solution in the z direction depends on the effective potential U_eff. Assuming that the transverse dimensions are much larger than the Fermi wavelength, the quantization in the transverse direction can be neglected and k_x, k_y are continuous. It is worth emphasizing that the total current given by Supplementary Equation (8) corresponds to the sum over the transversal modes (energy $\epsilon_\perp$) of the tunneling currents through

an effective barrier potential in the z direction U_{eff}. As explicitly written in Supplementary Equation (5), we are considering here the total potential including the potential barrier $U(z)$ and the biasing energy $U_{\text{bias}}(z, eV) = -eV(1-z/d)$, $U_{\text{eff}}(z) = U_{\text{bias}}(z, eV) = U(z) + U_{\text{bias}}(z, eV)$. The 3D transmission can be expressed as a function of the WKB transmission through the 1D effective potential:

$$T_{\text{3D}}(k_{\text{x}}, k_{\text{y}}, \epsilon) = T_{\text{WKB}}(\epsilon_{\parallel}, eV) = \exp\left\{-\frac{\sqrt{8m}}{\hbar}\int_{z_{\text{min}}(\epsilon_{\text{z}},eV)}^{z_{\text{max}}(\epsilon_{\text{z}},eV)} dz\sqrt{U(z) - eV\left(1-\frac{z}{d}\right) - \epsilon_{\parallel}}\right\}. \tag{5}$$

For free electron dispersions the transmission probability only depends on the total energy $\epsilon = \epsilon_{\text{x}} + \epsilon_{\text{y}} + \epsilon_{\parallel}$ and the magnitude of transverse wave vector $k_{\perp}$. The integration over $k_{\perp}$ is thus restricted to values which conserve ϵ and $(k_{\text{x}}, k_{\text{y}})$:

$$I(V) = \frac{2e}{h}\int_0^{+\infty} d\epsilon_{\parallel} \int_{k_{\text{x}}=-\infty}^{+\infty} \frac{dk_{\text{x}}}{2\pi/\sqrt{S}} \int_{k_{\text{y}}=-\infty}^{+\infty} \frac{dk_{\text{y}}}{2\pi/\sqrt{S}} T_{\text{WKB}}(\epsilon_{\parallel}, eV)\left[f(\epsilon) - f(\epsilon + eV)\right], \tag{6a}$$

$$= \frac{2e}{h}\frac{S}{(2\pi)^2}\int_0^{+\infty} d\epsilon_{\parallel} \int_{\text{k}_{\parallel}=0}^{+\infty} (2\pi)k_{\perp}dk_{\perp}\, T_{\text{WKB}}\left(\epsilon - \epsilon_{\perp}, eV\right)\left[f(\epsilon) - f(\epsilon + eV)\right], \tag{6b}$$

$$= \frac{2e}{h}\frac{S}{2\pi}\int_0^{+\infty} d\epsilon_{\parallel} \int_0^{+\infty} \frac{m}{\hbar^2} d\epsilon_{\perp}\, T_{\text{WKB}}(\epsilon_{\parallel}, eV)\left[f(\epsilon_{\parallel} + \epsilon_{\perp}) - f((\epsilon_{\parallel} + \epsilon_{\perp} + eV)\right]. \tag{6c}$$

(6d)

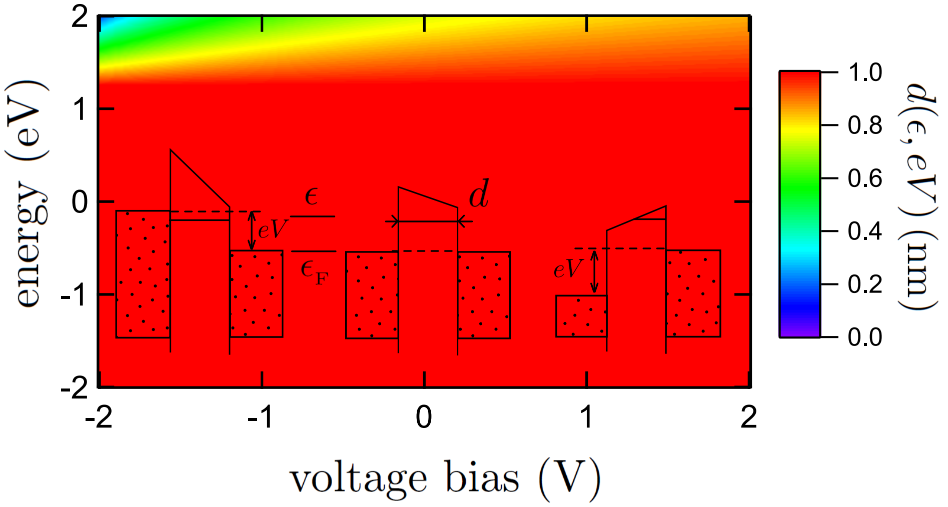

Supplementary Figure 4. Energy and voltage dependence of the barrier thickness d. The schematics allow to visualize the distortion of the barrier for $eV < 0$, $eV = 0$ and $eV > 0$.

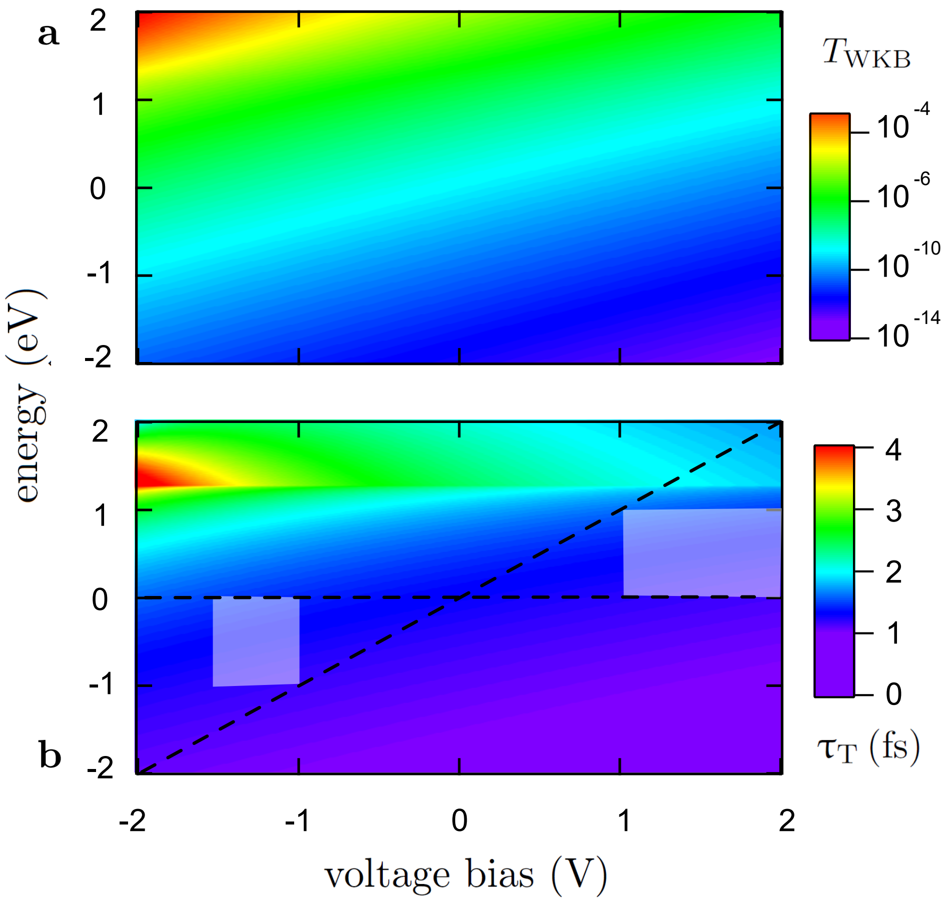

Supplementary Figure 5. **(a)** Color plot of the ratios of $T_{\rm WKB}(\epsilon, eV)$. Here, the energy ϵ is the energy measured from the Fermi energy $\epsilon_{\rm F}$. **(b)** Color plot of the ratios of $\tau_{\rm T}(\epsilon, eV)$. Shaded areas correspond to the voltage-energy range of interest (Figure 5b in the article).

The tunnel current I flowing from the left electrode to the right electrode then reads:

$$I(V) = \frac{2eM}{h} \int d\epsilon\, \mathcal{T}(\epsilon, eV)\, [f(\epsilon) - f(\epsilon + eV)]\,, \qquad (7)$$

where $M = \pi S/\lambda_F^2$ is the number of transversal modes of conduction contained in the tunnel junction area S. Instead of integrating over longitudinal energy $\epsilon_\parallel$, we can also integrate over the transversal one $\epsilon_\perp$ in order to keep the expression of the WKB transmission. We then recover a LB-like expression replacing the Fermi-Dirac distribution f by the pseudo-distribution $\tilde{f}(\epsilon_\parallel) \equiv \int_0^{+\infty} f(\epsilon_\parallel + \epsilon_\perp)\, \frac{d\epsilon_\perp}{\epsilon_{\rm F}} = -\frac{k_{\rm B}T}{\epsilon_{\rm F}} \log\left(f(\epsilon_\parallel)\right)$. The supplementary equation 6c gives :

$$I(V) = \frac{2eM}{h} \int d\epsilon_\parallel\, T_{\rm WKB}(\epsilon_\parallel, eV) \left[\tilde{f}(\epsilon_\parallel) - \tilde{f}(\epsilon_\parallel + eV)\right] \simeq \frac{2e^2M}{h} \overline{T}_{\rm WKB} V \qquad (8)$$

This expression is formally equivalent to the current LB formula of a single channel conductor and will be useful to get the estimation of the traversal-time in the 2D-tunnel junction. We then introduce the mean value of the transmission of the junction transmission channel:

$$\overline{T}_{\rm WKB}(\epsilon_\parallel, eV) = \frac{1}{M} \int_0^{\epsilon} \frac{d\epsilon_\perp}{\epsilon_{\rm F}} T_{\rm WKB}(\epsilon_\parallel, eV), \qquad (9)$$

where $T_{\rm WKB}(\epsilon_\parallel, eV)$ is the WKB transmission coefficient through a 1D potential barrier $U(z)$ and d is the thickness of the barrier. The biasing energy considers only the energy of the tunneling electron in the uniform electric field induced by the bias voltage, we have implicitly neglected the effects of space charge inside the barrier and image charge in the electrodes. In aluminum, the Fermi energy is $\epsilon_{\rm F} = 11.7\,$eV and the Fermi wavelength $\lambda_{\rm F} = 0.36\,$nm. Then, the

number of channels in the tunnel junction is $M \sim 2.4\times10^{11}$ and the average transmission in the considered voltage range is $\mathcal{T} < 1.7\times10^{-11}$. Here we model the barrier as a trapezoidal potential $U(z) = U + \Delta U(1/2 - z/d)$. The asymmetry ΔU of the trapezoidal barrier is obtained with the second order expansion of the normalized conductance [6]:

$$\frac{G(V)}{G(0)} = 1 + \frac{V}{V_1} + \left(\frac{V}{V_2}\right)^2 , \tag{10}$$

with $\Delta U/U = -3\sqrt{2}V_2/V_1$. The parabolic fit of data in the inset of Figure 2 in the article gives $V_1 \simeq -2.85\pm1\,\mathrm{V}$ and $V_2 \simeq 0.78\pm0.06\,\mathrm{V}$. We then deduce $\Delta U/U \simeq 1.1$. The values of $U \simeq 2.68\,\mathrm{eV}$ and $d \simeq 2\,\mathrm{nm}$ are estimated from the fit of the $I(V)$ characteristics. These values are obtained by considering the effective mass of electrons in the aluminum oxide AlO_x: $m = 0.38\times9.1\times10^{-31}$ kg [7]. We have checked that the charging effects in the barrier slightly change U and d of about 10%. The large value of the asymmetry $\Delta U/U$ can be attributed to the growth of aluminum film on different substrates (SiO_2/AlO_x). One has to keep in mind that the trapezoidal barrier model is a simplistic model which cannot fully describe our sample. The capacitance $\sim 0.5\,\mathrm{nF}$ of the tunnel junction is measured thanks to the cut-off frequency observed on the noise spectral density at low bias voltage. This value is in agreement with the thickness of the tunnel barrier: $C = \epsilon_\mathrm{r}\epsilon_0 S/d \simeq 0.43\,\mathrm{nF}$ where $\epsilon_\mathrm{r} = 9.8$ is the dielectric constant of alumina, ϵ_0 is the vacuum permittivity and S the surface of the junction.

Gauge invariance

The gauge transformation corresponds to the addition of a constant potential V_0 on both electrodes. It leads to the following transformations: $-eV(1-x/d) \to -eV(1-z/d) - eV_0$ for the biasing energy, $\mathcal{T}_\mathrm{WKB}(\epsilon, eV) \to \mathcal{T}_\mathrm{WKB}(\epsilon + eV_0, eV)$ for the WKB transmission coefficient and $f(\epsilon+eV) \to f(\epsilon + e(V+V_0))$ and $f(\epsilon) \to f(\epsilon + eV_0)$ for the Fermi-Dirac distributions in the electrodes. It is straightforward to check that Equations (3)(4a)(4b) in the article are invariant under these transformations.

Current noise spectral density at finite frequency for a 3D tunnel junction

As it has been shown in the article, the noise spectral density depends on the electrode where it is evaluated because of the energy and voltage dependent transmission ($S_\mathrm{LL} \neq S_\mathrm{RR} \neq -S_\mathrm{LR}$). It should also be stressed that, if we only consider the energy dependence of the transmission and omit its voltage dependence, the gauge invariance is violated and only one of the correlators satisfies the FDR, $S_\mathrm{LL} = S_\mathrm{ii}^{(\mathrm{FDR})}$ according to our choice of voltage biasing. For a 3D tunnel junction, Equation (3b) in the article has to be slightly modified to take into account the summation over the transversal modes:

$$\begin{aligned} S_\mathrm{LR}(eV, h\nu) = -\frac{2Me^2}{h}\int d\epsilon \int_0^\epsilon \frac{d\epsilon'}{\epsilon_\mathrm{F}} \sqrt{\mathcal{T}_\mathrm{WKB}(\epsilon', eV)\mathcal{T}_\mathrm{WKB}(\epsilon' - h\nu, eV)} \\ \times \left\{ f_\mathrm{L}(\epsilon)\left[1 - f_\mathrm{R}(\epsilon - h\nu)\right] + f_\mathrm{R}(\epsilon)\left[1 - f_\mathrm{L}(\epsilon - h\nu)\right]\right\}, \end{aligned} \tag{11}$$

whereas Eq. (3a) in the article remains unchanged considering the transmission given by Supplementary Equation (9). Thus, the total noise spectral density in the 3D tunnel junction reads:

$$\begin{aligned} S_\mathrm{ii}(eV, h\nu) = \frac{2Me^2}{h} \Bigg\{ &(1 + N(eV - h\nu)) \int d\epsilon \int_0^\epsilon \frac{d\epsilon_\perp}{\epsilon_\mathrm{F}} \left(\frac{\sqrt{\mathcal{T}_\mathrm{WKB}(\epsilon - \epsilon_\perp, eV)} + \sqrt{\mathcal{T}_\mathrm{WKB}(\epsilon - \epsilon_\perp + h\nu, eV)}}{2}\right)^2 \\ &\times \left[f(\epsilon) - f\left(\epsilon + e\left(V - \frac{h\nu}{e}\right)\right)\right] \\ &+ N(eV + h\nu) \int d\epsilon \int_0^\epsilon \frac{d\epsilon_\perp}{\epsilon_\mathrm{F}} \left(\frac{\sqrt{\mathcal{T}_\mathrm{WKB}(\epsilon - \epsilon_\perp, eV)} + \sqrt{\mathcal{T}_\mathrm{WKB}(\epsilon - \epsilon_\perp - h\nu, eV)}}{2}\right)^2 \\ &\times \left[f(\epsilon) - f\left(\epsilon + e\left(V + \frac{h\nu}{e}\right)\right)\right]\Bigg\}, \end{aligned} \tag{12}$$

As for the one-dimensional case, by using the expressions $f(\epsilon+\epsilon_0)\,(1-f(\epsilon)) = N(\epsilon_0)\,(f(\epsilon)-f(\epsilon+\epsilon_0))$ and $f(\epsilon)\,(1-f(\epsilon+\epsilon_0)) = (1+N(\epsilon_0))\,(f(\epsilon)-f(\epsilon+\epsilon_0))$ and by integrating over the transverse energy $\epsilon_\perp$ and using the mean transmission, we get:

$$\frac{M}{\epsilon_\mathrm{F}}\int_0^\infty d\epsilon_\perp d\epsilon_\mathrm{z} f(\epsilon+\epsilon_0)\,(1-f(\epsilon)) = N(\epsilon_0)\left(\tilde{f}(\epsilon)-\tilde{f}(\epsilon+\epsilon_0)\right) \tag{13}$$

and,

$$\frac{M}{\epsilon_\mathrm{F}}\int_0^\infty d\epsilon_\perp d\epsilon_\mathrm{z} f(\epsilon)\,(1-f(\epsilon+\epsilon_0)) = (1+N(\epsilon_0))\left(\tilde{f}(\epsilon)-\tilde{f}(\epsilon+\epsilon_0)\right). \tag{14}$$

Then, the noise spectral density is given by an integral over the transverse energy:

$$\begin{aligned} S_\mathrm{ii}(eV,h\nu) = \frac{2e^2}{h}\Bigg\{ &(1+N(eV-h\nu))\int d\epsilon_\mathrm{z}\left(\frac{\sqrt{T_\mathrm{WKB}(\epsilon_\mathrm{z},eV)}+\sqrt{T_\mathrm{WKB}(\epsilon_\mathrm{z}+h\nu,eV)}}{2}\right)^2\left[\tilde{f}(\epsilon_\mathrm{z})-\tilde{f}\left(\epsilon_\mathrm{z}+e\left(V-\frac{h\nu}{e}\right)\right)\right] \\ &+N(eV+h\nu)\int d\epsilon_\mathrm{z}\left(\frac{\sqrt{T_\mathrm{WKB}(\epsilon_\mathrm{z},eV)}+\sqrt{T_\mathrm{WKB}(\epsilon_\mathrm{z}-h\nu,eV)}}{2}\right)^2\left[\tilde{f}(\epsilon_\mathrm{z})-\tilde{f}\left(\epsilon_\mathrm{z}+e\left(V+\frac{h\nu}{e}\right)\right)\right]\Bigg\}, \end{aligned} \tag{15}$$

As for the expression of the current, this expression is formally equivalent to the noise spectral density of a single channel conductor with the WKB transmission $T_\mathrm{WKB}(\epsilon_\mathrm{z},eV)$.

Supplementary Figure 6 shows the theoretical noise spectral densities S_LL, S_RR and S_LR at $\lambda = hc/\nu = 1.3\,\mu\mathrm{m}$ using the parameters $(U,\Delta U,d)$ of the junction. S_LL and S_RR exhibit a strong dissymmetry revealing that energy relaxation occurs essentially in the left (*resp.* right) electrode for $eV<0$ (*resp.* $eV>0$) and can be interpreted as an electron-hole pairs recombination in the left (*resp.* right) electrode [8]. S_LR is more difficult to interpret and appears as an interference between the two former processes. Note that S_LR is almost proportional to S_LL (*resp.* S_RR) for $eV>0$ (*resp.* $eV>0$). It implies, because of the strong asymmetry, $S_\mathrm{LR}\simeq-\sigma(\nu)\,(S_\mathrm{LL}+S_\mathrm{RR})$ with $\sigma>0$ a factor of proportionality depending on the frequency ν (Supplementary Figure 6). The tunneling current is assumed to be constant in the barrier and given by the average current $I_\mathrm{T}=(I_\mathrm{L}-I_\mathrm{R})/2$ (see main text). The tunneling current noise spectral density is then:

$$S_\mathrm{TT}(eV,h\nu) = \frac{1}{4}\left(S_\mathrm{LL}+S_\mathrm{RR}-2S_\mathrm{LR}\right). \tag{16}$$

Although photon emission is due to the coupling to the fluctuations of the tunneling current S_TT, we can compare this quantity to the fluctuations of the accumulated charges on the electrodes of the junction related to $dQ/dt=(I_\mathrm{L}+I_\mathrm{R})/2$:

$$S_{\dot{\mathrm{Q}}\dot{\mathrm{Q}}}(eV,h\nu) = \frac{1}{4}\left(S_\mathrm{LL}+S_\mathrm{RR}+2S_\mathrm{LR}\right). \tag{17}$$

Supplementary Figure 6 and Supplementary Figure 7 show a significant difference between S_TT and $S_{\dot{\mathrm{Q}}\dot{\mathrm{Q}}}$. We also notice that $S_{\dot{\mathrm{Q}}\dot{\mathrm{Q}}}=0$ at zero frequency. The experimental data presented in the article in Fig. 4 falls on S_TT and are not in agreement with $S_{\dot{\mathrm{Q}}\dot{\mathrm{Q}}}$.

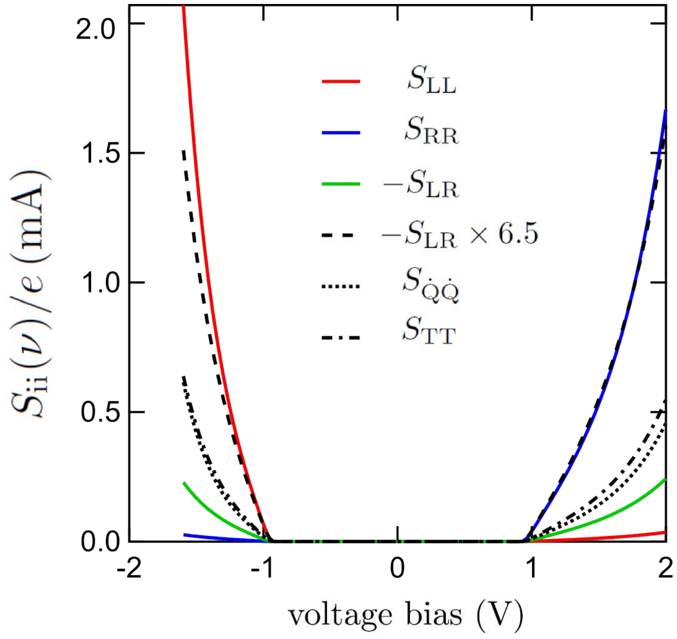

Supplementary Figure 6. Theoretical current noise spectral density $S_{\rm LL}$, $S_{\rm RR}$ and $S_{\rm LR}$ in the high frequency limit ($\lambda = 1.3\,\mu$m) for the trapezoidal barrier used to fit the experimental $I(V)$ characteristics.

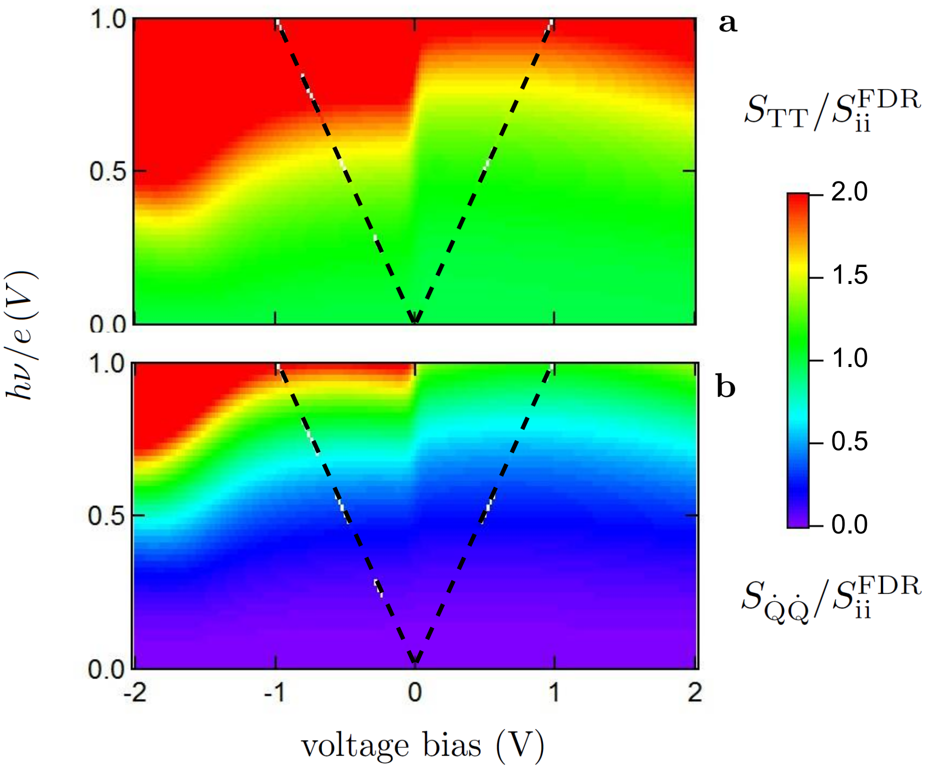

Supplementary Figure 7. **(a)** Color plot of the ratios $S_{\rm TT}/S_{\rm ii}^{\rm (FDR)} = (S_{\rm LL} + S_{\rm RR} - 2S_{\rm LR})/(4S_{\rm ii}^{\rm (FDR)})$. **(b)** Color plot of the ratios $S_{\dot{\rm Q}\dot{\rm Q}}/S_{\rm ii}^{\rm (FDR)} = (S_{\rm LL} + S_{\rm RR} + 2S_{\rm LR})/(4S_{\rm ii}^{\rm (FDR)})$. Dashed lines correspond to the cross-over $eV = \pm h\nu$.

Universal fluctuation-dissipation relation at zero frequency

We give here a derivation of the FDR at zero frequency using the steady state fluctuation theorem (SSFT). This theorem results in a generalization of the second law of thermodynamics and holds under very general hypothesis [10, 11]. We describe the electronic transport through the tunnel junction as a charge transfer where $\Gamma_{+/-}$ stands for the probability per unit time to transfer an electron from the left/right electrode to the right/left electrode. Note that no particular hypothesis is made on the transfer rates $\Gamma_{+/-}$. The resulting probability $p(q)$ to transfer a charge q during a tunneling event is given by:

$$p(q) = \left[1 - (\Gamma_+ + \Gamma_-)\delta t\right]\, \delta(q) + \Gamma_+ \delta t\, \delta(q-e) + \Gamma_- \delta t\, \delta(q+e), \tag{18}$$

where δt is the characteristic time of the tunneling event. In the long time limit ($\Delta t = N\delta t \to +\infty$), the charge Q transferred through the junction is the sum of N independent random variables $Q = \sum_{i=1}^{N} q_i$ and its distribution probability reads:

$$P(Q) = \int \prod_{i=1}^{N} dq_i\, p(q_i) \delta\left(Q - \sum_{i=1}^{N} q_i\right). \tag{19}$$

Let's introduce the moment generating function $\chi_{\mathrm{Q}}(\lambda)$ which offers a convenient way to characterize the distribution function P:

$$\chi_{\mathrm{Q}}(\lambda) = \int dQ\, P(Q) e^{i\lambda Q} = \left((1 - (\Gamma_+ + \Gamma_-)\delta t) + \Gamma_+ \delta t e^{i\lambda e} + \Gamma_- \delta t e^{-i\lambda e}\right)^N, \tag{20}$$

which becomes in the tunneling limit ($\Gamma_{+/-}\delta t \ll 1$):

$$\chi_{\mathrm{Q}}(\lambda) \simeq 1 - N\delta t\left[(\Gamma_+ + \Gamma_-) - \Gamma_+ \delta t e^{i\lambda e} - \Gamma_- \delta t e^{-i\lambda e}\right]. \tag{21}$$

The SSFT states that $P(Q)/P(-Q) = e^{-QV/k_B T}$ for a voltage biased tunnel junction. We thus deduce:

$$\chi_{\mathrm{Q}}(\lambda) = \chi_{\mathrm{Q}}\left(-\lambda - i\frac{V}{k_{\mathrm{B}}T}\right), \tag{22}$$

allowing to deduce a detailed balance relation between the transfer rate coefficients $\Gamma_{+/-}$:

$$\frac{\Gamma_+}{\Gamma_-} = \exp\left(-\frac{eV}{k_{\mathrm{B}}T}\right). \tag{23}$$

The mean value of the current $\langle I\rangle = \langle Q\rangle/\Delta t$ and its fluctuations $\langle \Delta I^2\rangle = \langle \Delta Q^2\rangle/\Delta t^2$ are then given by the first two terms of the Taylor expansion of the generating function

$$\langle I\rangle = e\left(\Gamma_+ - \Gamma_-\right), \tag{24a}$$
$$\langle \Delta I^2\rangle = e^2\left(\Gamma_+ + \Gamma_-\right)\Delta f. \tag{24b}$$

where $\Delta f = 1/\Delta t \to 0$ is the frequency bandwidth of the measurement. We finally obtain the FDR:

$$S_{\mathrm{ii}}(eV, h\nu = 0) = \frac{\langle \Delta I^2\rangle}{\Delta f} = \frac{eI(V)}{\tanh\left(eV/2k_{\mathrm{B}}T\right)}. \tag{25}$$

SUPPLEMENTARY NOTE 4
TRAVERSAL TIME IN A 3D TUNNEL JUNCTION

The time for an electron to cross a 1D barrier is defined as the traversal-time [9]:

$$\tau_{\mathrm{T}}(\epsilon, eV) = \int_{z_{\min}(eV,\epsilon)}^{z_{\max}(eV,\epsilon)} \sqrt{\frac{m}{2\left(U(z) + eV\left(1 - \frac{z}{d}\right) - \epsilon\right)}}\, dz. \tag{26}$$

By considering the energy derivative of Supplementary Equation (5), we get:

$$\frac{\partial \log\left(T_{\mathrm{WKB}}\right)}{\partial \epsilon} = \frac{2\tau_{\mathrm{T}}}{\hbar}. \tag{27}$$

Thus the traversal-time is simply related to the WKB transmission:

$$\frac{T_{\mathrm{WKB}}(\epsilon_2, eV)}{T_{\mathrm{WKB}}(\epsilon_1, eV)} = \exp\left\{\frac{2}{\hbar}\int_{\epsilon_1}^{\epsilon_2} \tau_{\mathrm{T}}(\epsilon', eV)\, d\epsilon'\right\}. \tag{28}$$

Supplementary Figure 5 shows these two quantities as a function of the bias voltage eV and the energy ϵ of the incoming electron for the trapezoidal barrier previously defined. If the transmission varies over 8 order of magnitude (Supplementary Figure 5a), the traversal-time varies by only 50% in the range of interest (see shaded areas in Supplementary Figure 5b).
As we have already seen, the tunneling in the 3D tunnel junction is described by the tunneling of transversal modes through the effective potential $U_{\mathrm{eff}} = \epsilon_\perp + \frac{\hbar^2 k_{parallel}^2}{2m} + U(z)$. The traversal-time corresponding to the transversal mode of energy ϵ is then $\tau_{\mathrm{T}}(\epsilon - \epsilon_\perp)$. Thus the traversal-time in the full energy range is the same for the 1D and the 3D tunnel junction. Finally, according to Supplementary Equation (12), assuming that τ_{T} is rather constant on the energy scale $[\epsilon_{\mathrm{F}}, \epsilon_{\mathrm{F}} + h\nu]$ and that transversal channels of energy $\epsilon_{\mathrm{x}} + \epsilon_{\mathrm{y}} > h\nu$ have a negligible transmission, we recover Equation (13) of the Method.

SUPPLEMENTARY NOTE 5
VALIDITY OF THE FLUCTUATION-DISSIPATION RELATION AT FINITE FREQUENCY

As it has been shown in the article for a 1D channel, $S_{\mathrm{ii}} = S_{\mathrm{ii}}^{(\mathrm{FDR})}$ only if $\sqrt{\mathcal{T}(\epsilon, eV)} + \sqrt{\mathcal{T}(\epsilon \pm h\nu, eV)} = 2\sqrt{\mathcal{T}(\epsilon, eV \mp h\nu)}$. According to Supplementary Equation (12), this condition remains valid for the multi-channels tunnel junction. It means that $\mathcal{T}(\epsilon, eV) = t(\epsilon, eV)^2$ with:

$$t(x, y) + t(x + z, y) = 2t(x, y - z) \quad \forall x, y, z. \tag{29}$$

By considering $z = y - x$ and $u(x) = t(x, x)$, a straightforward calculation gives:

$$t(x, y) = u(x) - \frac{u(y)}{2} \quad \text{with} \quad u(x + y) = u(x) + u(y). \tag{30}$$

Thus, u is a linear function and the transmission is of the form $\mathcal{T}(\epsilon, eV) = \mathcal{T}_0\left(1 + (\epsilon - eV/2)/\epsilon_0\right)^2$. This form is a good approximation of the tunneling transmission only at small bias voltage $eV \ll U$. For a single channel conductor, using directly the WKB transmission, we get:

$$\mathcal{T}_0 = \exp\left(-\sqrt{\frac{8mUd^2}{\hbar^2}}\right) \quad \text{and} \quad \epsilon_0 = \sqrt{\frac{2\hbar^2 U}{md^2}}. \tag{31}$$

For a multi-channel tunnel junction, ϵ_0 remains unchanged, while:

$$\mathcal{T}_0 = M\frac{\epsilon_0}{\epsilon_{\mathrm{F}}}. \tag{32}$$

However, even if the transmission is not a quadratic function of $\epsilon - eV/2$, the ratio $S_{\mathrm{ii}}/S_{\mathrm{ii}}^{(\mathrm{FDR})}$ is nearly voltage independent for $h\nu < 1\,\mathrm{eV}$ (Supplementary Figure 7). This can be misleading. It indeed means that, even if $S_{\mathrm{ii}}^{(\mathrm{FDR})}$ could approximatively explain the voltage dependence of the emitted light power P_ν, the radiation impedance would be overestimated because S_{ii} is underestimated. In reference [12], Roussel *et al.* show the validity of the FDR provided few hypothesis. They use the non-equilibrium Kubo formula [13–15]:

$$S_{\mathrm{ii}}(eV, -h\nu) - S_{\mathrm{ii}}(eV, h\nu) = 2h\nu \mathrm{Re}\left(G(eV, h\nu)\right), \tag{33}$$

combined with the photon-assisted tunneling formula,

$$\mathrm{Re}\left(G(eV, h\nu)\right) = e\frac{I(eV + h\nu) - I(eV - h\nu)}{2h\nu}, \tag{34}$$

where $G(eV, h\nu)$ is the non-equilibrium ac conductance measured at frequency ν for a dc voltage bias V. However, Supplementary Equation (34) does not hold for a voltage dependent transmission which is responsible for the FDR violation [15]. It is also important to notice that $G(eV, h\nu)$ is not well defined at optical frequencies because of transversal dependence of the ac voltage related to the SPP excitation on the electrode of the tunnel junction.

We also may ask questions about the validity of the LB approach at optical frequencies. We only use it to calculate the tunneling current which couples to the electric field in the barrier. The LB formalism assumes that the electron wave vector is constant, equals to the Fermi wave vector k_{F}. This assumption is valid since we are considering electrons with energy ϵ close to the Fermi energy ($\epsilon_{\mathrm{F}} = 11.7\,\mathrm{eV}$ in aluminum). At optical frequencies ν, $|\epsilon - \epsilon_{\mathrm{F}}| \sim h\nu \sim 1\,\mathrm{eV}$ and the current becomes position dependent on a typical length scale $l \sim \frac{2\epsilon_{\mathrm{F}}}{h\nu}\lambda_{\mathrm{F}} \sim 7\,\mathrm{nm}$ which remains larger than the electrode thickness.

SUPPLEMENTARY NOTE 6
SURFACE PLASMON POLARITON MODES IN THE TUNNEL JUNCTION - RADIATION IMPEDANCE

We consider here a simple tunnel junction made of two thick metallic layers with a total thickness a separated by a thin layer of insulator. We can therefore distinguish two kinds of surface plasmon polariton (SPP) modes, the fast mode localized at the surfaces of the electrodes and the slow mode localized inside the tunneling barrier of thickness $d \ll a$. However, only the fast mode at the vacuum interface is coupled to the propagating mode in the sapphire substrate (inset in Supplementary Figure 8). In the following, we then model the junction by a single metallic film of thickness a. In first approximation, we consider the dispersion relation of a semi-infinite metallic layer ($a \to +\infty$) [16]:

$$k_{\mathrm{SPP},\infty} = \frac{\omega}{c}\sqrt{\frac{\epsilon_1\epsilon_2}{\epsilon_1 + \epsilon_2}}, \tag{35}$$

where $\epsilon_1 = 1 - (\omega_{\mathrm{p}}/\omega)^2 - i\gamma_{\mathrm{p}}\omega_{\mathrm{p}}^2/\omega^3$ is the Drude dielectric constant of the metal described by the plasma frequency ω_{p} and the damping term γ_p. $\epsilon_2 = 1$ is the dielectric constant of the vacuum. In aluminum, reference [17] gives $\omega_{\mathrm{p}} = 14.7\,\mathrm{eV}$, $\gamma_{\mathrm{p}} = 80\,\mathrm{meV}$ and $\delta_{\mathrm{p}} = 12.7\,\mathrm{nm}$. Supplementary Figure 8 shows the theoretical expectation of Supplementary Equation (35). For a thin film ($a < \delta_{\mathrm{p}}$), k_{SPP} is not real anymore and the fast SPP mode can leak in the substrate. In the low frequency limit $\omega \ll \omega_{\mathrm{p}}$, the fast SPP mode reduces to $k_{\mathrm{SPP}} = \omega/c + \delta k_{\mathrm{SPP}}$ with $\delta k_{\mathrm{SPP}} \ll \omega/c$ and the emission occurs in the substrate at the specific angle $\theta_{\mathrm{p}} \simeq \arcsin 1/n$ such that $k_{\mathrm{SPP}} = n\omega/c \sin\theta_{\mathrm{p}}$. It corresponds to the crossing between the light-line and the dispersion relation of the SPP mode (see dashed lines in Supplementary Figure 8).

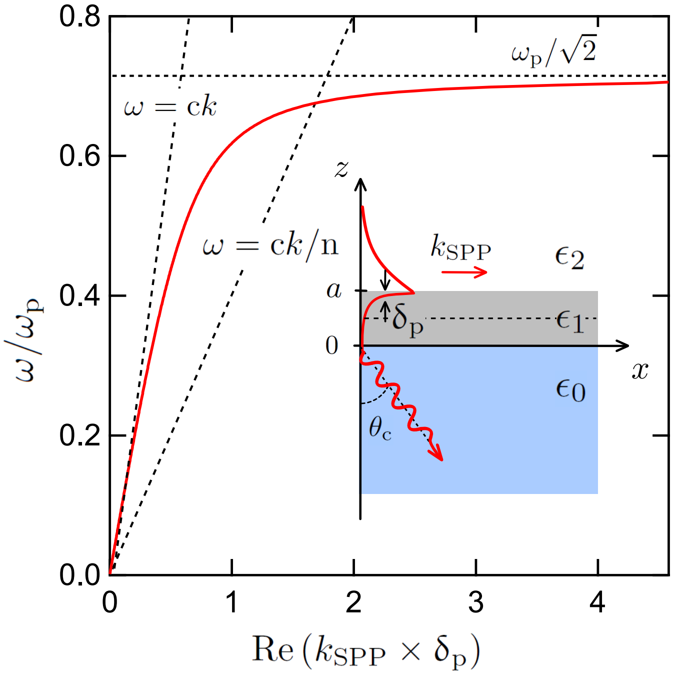

Supplementary Figure 8. Dispersion relation of the SPP mode at the vacuum interface. Dashed lines correspond to dispersion relations in vacuum ($k = \omega/c$) and sapphire ($k = n\omega/c$) respectively. *Inset*: geometry of the tunnel junction in contact with its sapphire substrate. The Kretschmann geometry enables the leakage of the SPP in the substrate at specific angle θ_p. Dashed line corresponds to the tunneling barrier.

Coupling in the Kretschmann configuration

We now consider a thin metallic layer of thickness a deposited on a substrate characterized by a dielectric constant $\epsilon_0 = n^2$ to evaluate the leakage radiation (inset in Supplementary Figure 8). We can assume that the thickness d of the tunneling barrier has no effect on the field in the metallic electrodes since $d \ll a$. The z component of the electric field in the junction is expressed by:

$$E_0^+ \exp(ik_{z0}z) + E_0^- \exp(-ik_{z0}z) \quad \text{for} \quad z < 0, \tag{36a}$$
$$E_1^+ \exp(ik_{z1}z) + E_1^- \exp(-ik_{z1}z) \quad \text{for} \quad 0 < z < a, \tag{36b}$$
$$E_2^+ \exp(ik_{z2}z) \quad \text{for} \quad a < z. \tag{36c}$$

with $\mathrm{Im}(k_{z,i}) > 0$. At the lowest non-trivial order in δk_{SPP}:

$$k_{z0} \simeq \frac{\omega}{c}\sqrt{n^2-1}, \tag{37a}$$
$$k_{z1} \simeq i\frac{\omega_p}{c}, \tag{37b}$$
$$k_{z2} \simeq \sqrt{-2\delta k_{\mathrm{SPP}}\frac{\omega}{c}}. \tag{37c}$$
$$\tag{37d}$$

By implementing the boundary conditions of continuity of the electric and magnetic field parallel to the surface, we get:

$$\frac{E_0^+}{E_2^+} = \frac{1}{2\sqrt{1-\beta^2}}\left\{\left(\frac{\epsilon_2}{\epsilon_0} + \frac{k_{z2}}{k_{z0}}\right) + \beta\left(\frac{\epsilon_1}{\epsilon_0}\frac{k_{z2}}{k_{z1}} + \frac{k_{z1}}{k_{z0}}\frac{\epsilon_2}{\epsilon_1}\right)\right\}, \tag{38}$$

with $\beta = -\tanh(ik_{z1}a)$. The dispersion relation $k_{\rm SPP}(\omega) = \omega/c + \delta k_{\rm SPP}$ is then solution of equation $E_0^+(k_{\rm SPP}) = 0$ which gives at the first non-trivial order:

$$\delta k_{\rm SPP} \simeq \frac{1}{2\delta_{\rm p}}\left(\frac{\omega}{\omega_{\rm p}}\right)^3\left\{1 - 2i\frac{\beta^2-1}{\beta}\frac{{\rm n}^2}{\sqrt{{\rm n}^2-1}}\left(\frac{\omega}{\omega_{\rm p}}\right)\right\} \tag{39}$$

Supplementary Figure 9 shows $\delta k_{\rm SPP}$ as a function of frequency in the low frequency limit $\omega \ll \omega_{\rm p}$. Its inset compares the coupling length ${\rm Im}(\delta k_{\rm SPP}^{-1})$ for different thickness to the Joule dissipation length:

$${\rm Im}(\delta k_{\rm SPP,\infty}^{-1}) \simeq \frac{1}{2}\frac{\gamma_{\rm p}}{c}\left(\frac{\omega}{\omega_{\rm p}}\right)^2. \tag{40}$$

It confirms that radiative damping is dominating for our experimental parameters $a = 10\,{\rm nm}$ and $0.064 < \omega/\omega_{\rm p} < 0.1$.

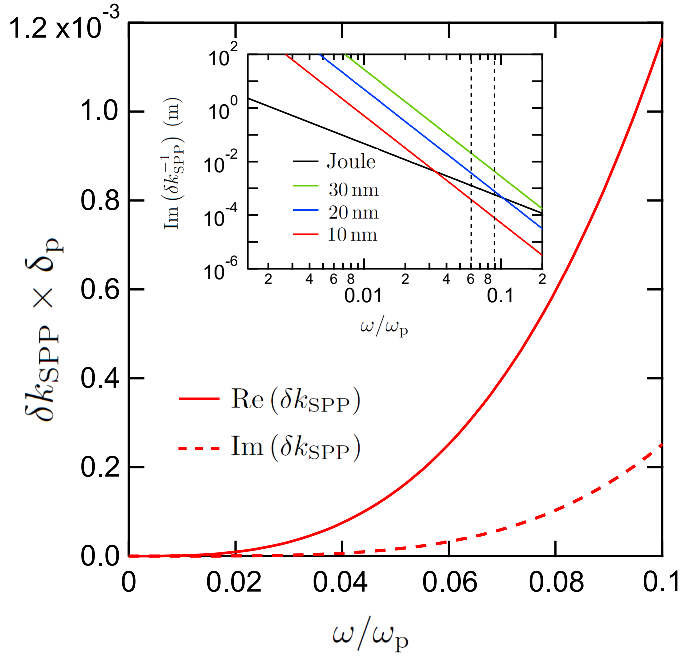

Supplementary Figure 9. Dispersion relation $\delta k_{\rm SPP} = k_{\rm SPP} - \omega/c$ in the low frequency limit $\omega \ll \omega_{\rm p}$. *Inset*: coupling length ${\rm Im}(\delta k_{\rm SPP}^{-1})$ in the Kretschmann configuration.

Electric and magnetic fields in the tunnel junction

Supplementary Figure 10 shows the profile of the electric $(E_x, 0, E_z)$ and magnetic $(0, H_y, 0)$ fields components at $\omega/\omega_{\rm p} = 0.064$. $E_{\rm x}$ and $H_{\rm y}$ are continuous whereas $E_{\rm z}$ exhibits discontinuities. The $z-$component of the electric field inside the tunnel barrier can be considered constant and is given at the third order in β by:

$$\frac{E_{\rm T}}{E_2^+} = \frac{\epsilon_1}{\epsilon_0}E_{z1}\left(\frac{a}{2}\right) = \frac{1}{\sqrt{2}n^2}\left(1 - \frac{\beta^2}{8} + O(\beta^4)\right), \tag{41}$$

where $\epsilon_0 = n^2$ is also the dielectric constant of the alumina Al_2O_3 which is the same as sapphire. Note that the mode in the substrate is oscillating due to the radiative leakage of the SPP in the Kretschmann configuration.

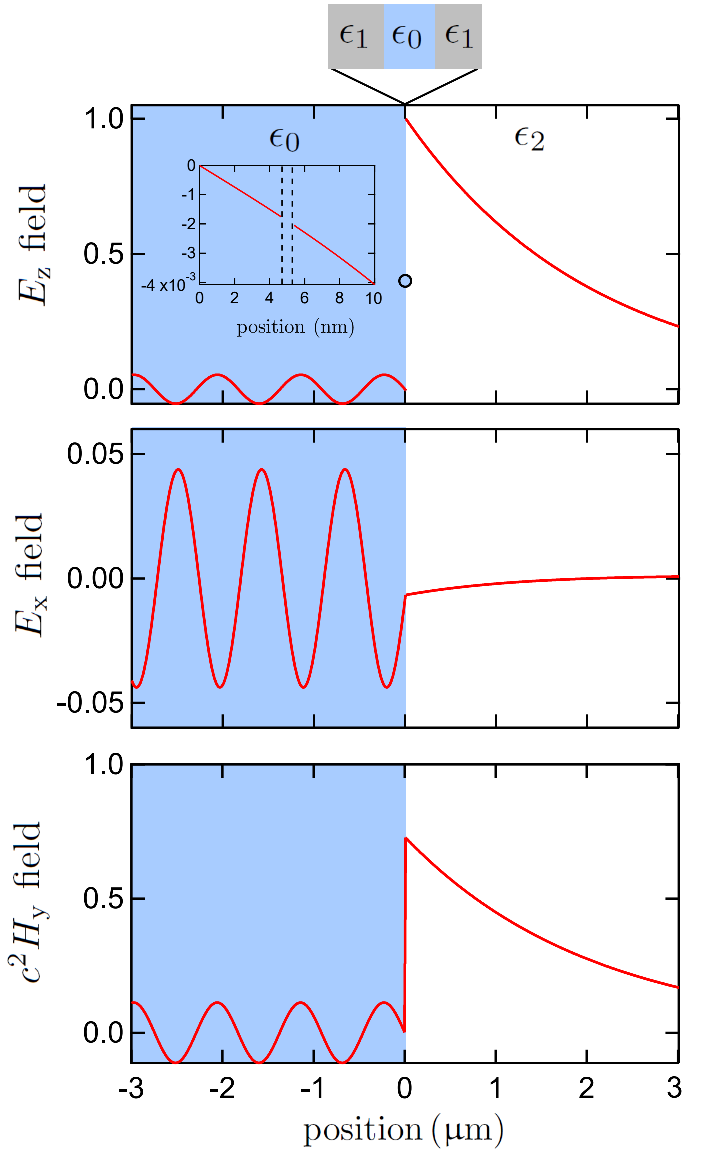

Supplementary Figure 10. Electric $(E_\mathrm{x}, 0, E_z)$ and magnetic $(0, H_\mathrm{y}, 0)$ fields of the $\lambda = 1.3\,\mu\mathrm{m}$ SPP fast mode. They are normalized with respect to the field E_z at position $z = d$. The blue circle corresponds to the value of the field in the thin $(d \sim 2\,\mathrm{nm})$ Al_2O_3 layer which corresponds to the tunnel barrier. *Inset of the upper graph*: $z-$component of the electric field in the electrodes.

Radiation impedance in the low frequency limit $\omega \ll \omega_\mathrm{p}$

The Laks-Mills theory of light emission in a tunnel junction gives a radiated spectral power as a function of the two point noise spectral density $S_\mathrm{ii}(z, z')$ and the $z-$component of the electric field inside the tunneling barrier [18–20]

$$P_\nu = Z_\mathrm{vac} \int_0^{2\pi} d\varphi \int_0^{\pi/2} d\theta \, \frac{\omega^2 \sin^3\theta}{4\pi^2 c^2} \int_0^d dz\, dz' \, \frac{E_{z1}(z) E^\star_{z1}(z')}{\left| E_0^+(k_x = n\frac{\omega}{c}\sin\theta)\right|^2} S_\mathrm{ii}(z, z'), \tag{42}$$

where $Z_\mathrm{vac} = \sqrt{\mu_0/\epsilon_0} \simeq 376.7\,\Omega$ is the vacuum impedance. By assuming a position independent electric field $(d \ll \delta_\mathrm{p})$ and a position independent tunneling current $I_\mathrm{T} = (I_\mathrm{L} - I_\mathrm{R})/2$ in the oxide barrier, we get the radiation impedance:

$$\mathcal{R} = Z_\mathrm{vac} \int_0^{\pi/2} d\theta \, \frac{\omega^2 \sin^3\theta}{2\pi c^2} d^2 \left| \frac{E_T}{E_0^+(n\frac{\omega}{c}\sin\theta)} \right|^2 . \tag{43}$$

In the low frequency limit $\omega \ll \omega_\mathrm{p}$, according to Eqs. (37a)(37b)(37c) and Supplementary Equation (38), the θ-dependence in E_0^+ only appears in $k_{z2} = \frac{\omega}{c}\sqrt{1-n^2\sin^2\theta}$ whereas $k_{z0} = \frac{\omega}{c}\sqrt{n^2-1}$ and $k_{z1} = i\omega_\mathrm{p}/c$. The integrand in Supplementary Equation (43) is thus dominated by its value in the region close to the pole

$$k_{z2,c} \simeq \sqrt{-\frac{2\omega}{c}\delta k_\mathrm{SPP}} \simeq \frac{1}{\beta\delta_\mathrm{p}}\left(\frac{\omega}{\omega_\mathrm{p}}\right)^2\left\{\frac{\beta^2-1}{\beta}\frac{n^2}{\sqrt{n^2-1}}\left(\frac{\omega}{\omega_\mathrm{p}}\right)+i\right\}, \tag{44}$$

when k_{z2} follows the contour $\frac{\omega}{c}\sqrt{1-n^2\sin^2\theta}$ with $\theta \in [0,\pi/2]$ (inset of Supplementary Figure 11). Substituting Eqs. (38) and (41) in Supplementary Equation (43), the lorentzian approximation of the integrand leads to the radiation impedance in the low frequency limit $\omega \ll \omega_\mathrm{p}$:

$$\mathcal{R}(\nu=\omega/2\pi) \simeq \frac{1}{\beta n^5}\left(\frac{d}{\delta_\mathrm{p}}\right)^2\left(\frac{\omega}{\omega_\mathrm{p}}\right)^3 Z_\mathrm{vac}, \tag{45}$$

corresponding to a directed emission at angle θ_p with:

$$\sin\theta_\mathrm{p} \simeq \frac{1}{n}\left\{1+\frac{1}{2\beta^2}\left(\frac{\omega}{\omega_\mathrm{p}}\right)^2\right\}. \tag{46}$$

Note that the angle of emission is slightly greater than the angle of total internal reflection of the flat substrate which explains the role of the conical prism. The radiation impedance due to the coupling between the tunneling current and the electromagnetic field is estimated at $\mathcal{R}(\nu) \sim 0.5\,\mathrm{m}\Omega$ for $\lambda = 1\,\mu\mathrm{m}$. We can also estimate the radiation impedance due the coupling of the current in the electrodes but the screening factor $(n\omega/\omega_\mathrm{p})^2 \sim 10^{-2}$ of the electric field in the metal leads to $\mathcal{R}(\nu) \sim 1\,\mu\Omega$ which disagrees with experiment. Note that our calculation neglects the Drude dissipation compared to the plasmon leakage in the substrate.

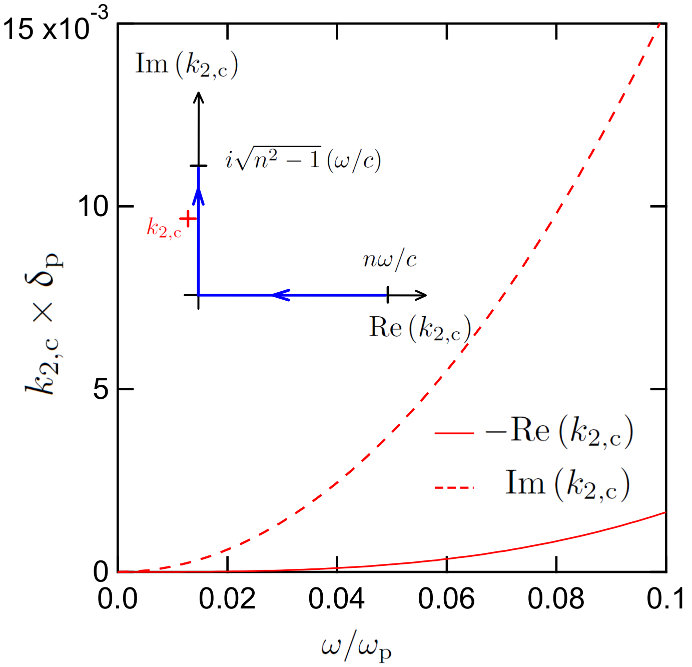

Supplementary Figure 11. Frequency dependence of the pole $k_{z2,c}$ in the low frequency limit $\omega \ll \omega_\mathrm{p}$. *Inset*: illustration of the integration contour used to calculate the radiation impedance.

Photon emission efficiency in a metallic tunnel junction

The emission efficiency is usually defined by an electron to photon conversion rate:

$$\frac{\int_0^{+\infty} P_\nu/(h\nu)d\nu}{I/e}. \tag{47}$$

However, as explained in the article, it is more relevant to define it with respect to the Joule power $P_\mathrm{J} = V \times I$ dissipated in the tunnel junction:

$$\eta(V) = \frac{\int_0^{+\infty} P_\nu d\nu}{V \times I(V)}. \tag{48}$$

Supplementary Figure 12 shows the theoretical current noise spectral density for different bias voltages. The junction is characterized by the set of parameters $(U, \Delta U, d)$ defined in Supplementary Note 3. It exhibits the cross-over at $h\nu = eV$ as expected. It enables to numerically calculate the efficiency η and demonstrate the relationship between the efficiency and the ratio $\mathcal{R}/R_\mathrm{K}$ (inset of Supplementary Figure 12):

$$\eta(V) \sim \eta_0 \frac{\mathcal{R}\left(\frac{eV}{h}\right)}{R_\mathrm{K}}, \tag{49}$$

where $\eta_0 \simeq 0.047$ in the low frequency limit $\omega \ll \omega_\mathrm{p}$ where the radiation impedance is given by Supplementary Equation (45). Note that η_0 is voltage dependent at low bias voltage giving rise to an increased efficiency. This is an artifact due to the black body radiation which are always emitting even at zero bias voltage. η_0 is a constant weakly dependent on the details of the barrier and depends mainly on the frequency dependence of the radiation impedance. Its numerical value is indeed close to $\eta_0 = 1/20$ found for a tunnel junction with constant transmission at zero temperature in the low frequency limit $\omega \ll \omega_\mathrm{p}$:

$$\eta = \frac{\int_0^{+\infty} \mathcal{R}(\nu) e I(V) \left(1 - (h\nu/eV)\right) d\nu}{V \times I(V)} = \frac{1}{20} \frac{\mathcal{R}\left(\frac{eV}{h}\right)}{R_\mathrm{K}}. \tag{50}$$

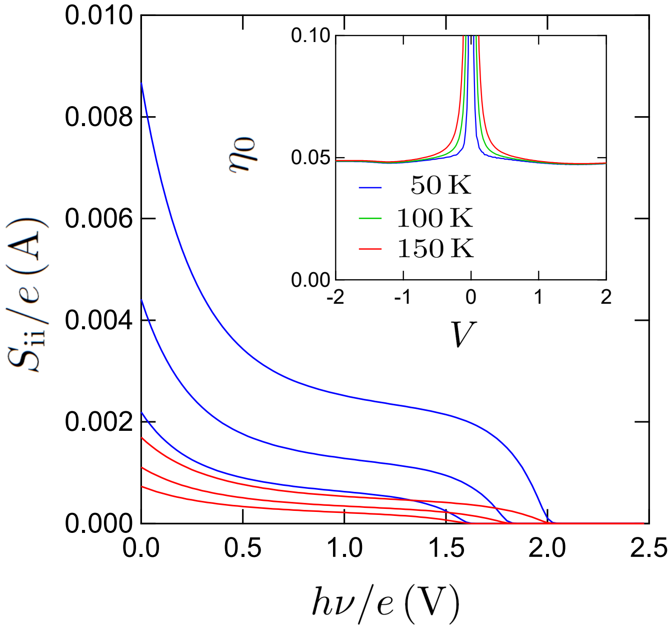

Supplementary Figure 12. Theoretical current noise spectral density for different bias voltage. Red (*resp.* Blue) lines corresponds to positive (*resp.* negative) voltages 2, 1.8 and 1.6 V. *Inset*: Voltage dependence of $\eta_0 = \eta \times (R_\mathrm{K}/\mathcal{R})$ for different temperatures in the low frequency limit $\omega \ll \omega_\mathrm{p}$.

SUPPLEMENTARY REFERENCES

[1] Kretschmann, E. Decay of non radiative surface plasmons into light on rough silver films. comparison of experimental and theoretical results. *Optics Communications* **6**, 185 – 187 (1972).

[2] Patiño, E. J. & Kelkar, N. G. Experimental determination of tunneling characteristics and dwell times from temperature dependence of al/al2o3/al junctions. *Applied Physics Letters* **107**, 253502 (2015).
[3] Lin, Z., Zhigilei, L. V. & Celli, V. Electron-phonon coupling and electron heat capacity of metals under conditions of strong electron-phonon nonequilibrium. *Phys. Rev. B* **77**, 075133 (2008).
[4] Simmons, J. G. Generalized thermal J-V characteristic for the electric tunnel effect. *J. Appl. Phys.* **35**, 2655–2658 (1964).
[5] Hansen, K. & Brandbyge, M. Current-voltage relation for thin tunnel barriers: Parabolic barrier model. *Journal of Applied Physics* **95**, 3582–3586 (2004).
[6] Brinkman, W. F., Dynes, R. C. & Rowell, J. M. Tunneling conductance of asymmetrical barriers. *Journal of Applied Physics* **41**, 1915–1921 (1970).
[7] Zemanová Diešková, M., Ferretti, A. & Bokes, P. Tunneling through Al/AlO$_x$/Al junction: Analytical models and first-principles simulations. *Phys. Rev. B* **87**, 195107 (2013).
[8] Zamoum, R., Lavagna, M. & Crépieux, A. Nonsymmetrized noise in a quantum dot: Interpretation in terms of energy transfer and coherent superposition of scattering paths. *Phys. Rev. B* **93**, 235449 (2016).
[9] Büttiker, M. & Landauer, R. Traversal time for tunneling. *Phys. Rev. Lett.* **49**, 1739–1742 (1982).
[10] Evans, D. J., Cohen, E. G. D. & Morriss, G. P. Probability of second law violations in shearing steady states. *Phys. Rev. Lett.* **71**, 2401–2404 (1993).
[11] Esposito, M., Harbola, U. & Mukamel, S. Fluctuation theorem for counting statistics in electron transport through quantum junctions. *Phys. Rev. B* **75**, 155316 (2007).
[12] Roussel, B., Degiovanni, P. & Safi, I. Perturbative fluctuation dissipation relation for nonequilibrium finite-frequency noise in quantum circuits. *Phys. Rev. B* **93**, 045102 (2016).
[13] Kubo, R. Statistical-mechanical theory of irreversible processes. i. general theory and simple applications to magnetic and conduction problems. *Journal of the Physical Society of Japan* **12**, 570–586 (1957).
[14] Safi, I. & Sukhorukov, E. V. Determination of tunneling charge via current measurements. *EPL (Europhysics Letters)* **91**, 67008 (2010).
[15] Gavish, U., Imry, Y. & Levinson, Y. Quantum noise, detailed balance and kubo formula in nonequilibrium quantum systems Preprint at https://arxiv.org/abs/cond-mat/0211681 (2002)
[16] Maier, S. A. *Plasmonics: Fundamentals and Applications* (Springer Science & Business Media).
[17] Ordal, M. A., Bell, R. J., Alexander, R. W., Long, L. L. & Querry, M. R. Optical properties of fourteen metals in the infrared and far infrared: Al, Co, Cu, Au, Fe, Pb, Mo, Ni, Pd, Pt, Ag, Ti, V and W. *Appl. Opt.* **24**, 4493–4499 (1985).
[18] Laks, B. & Mills, D. L. Photon emission from slightly roughened tunnel junctions. *Phys. Rev. B* **20**, 4962–4980 (1979).
[19] Laks, B. & Mills, D. L. Light emission from tunnel junctions: The role of the fast surface polariton. *Phys. Rev. B* **22**, 5723–5729 (1980).
[20] Ushioda, S., Rutledge, J. E. & Pierce, R. M. Theory of prism-coupled light emission from tunnel junctions. *Phys. Rev. B* **34**, 6804–6812 (1986).
